# Breastfeeding in Hospitals: Factors Influencing Maternal Choice in Italy

**DOI:** 10.3390/ijerph17103575

**Published:** 2020-05-20

**Authors:** Rosalia Ragusa, Gabriele Giorgianni, Marina Marranzano, Salvatore Cacciola, Valentina Lucia La Rosa, Alessandra Giarratana, Valentina Altadonna, Vincenzo Guardabasso

**Affiliations:** 1 Health Technology Assessment Committee, A.O.U. Policlinico V. Emanuele, 95123 Catania, Italy; 2 School of Specialization in Hygiene, University of Catania, 95123 Catania, Italy; giorgiangab@gmail.com (G.G.); alessandra.giarratana@gmail.com (A.G.); altavalen@gmail.com (V.A.); 3 Department of Advanced Medical, Surgical and Advanced Sciences, University of Catania, 95123 Catania, Italy; marranz@unict.it; 4 Health Education Unit, 95124 Catania, Italy; scacciola59@gmail.com; 5 Department of Educational Sciences, University of Catania, 95124 Catania, Italy; valarosa@unict.it; 6 Research Promotion Office, A.O.U. Policlinico V. Emanuele, 95123 Catania, Italy; guardabasso@policlinico.unict.it

**Keywords:** breastfeeding, maternal education, nursing

## Abstract

Monitoring the prevalence of breastfeeding is one of the actions provided for in Italian National Health System. This study aims to observe the prevalence of breastfeeding in a representative set of birthing hospitals in the province of Catania, in Sicily, Italy, to assess the factors influencing women in their decisions to breastfeed during hospitalization after delivery. We conducted an observational study on 3813 questionnaires administered to mothers of newborns during their hospital stay from the years 2016 to 2018 in eight hospitals of various types. The average maternal age was 31.3 years ± 5.8. Sixty-nine percent of women did not attend a prenatal course. From childbirth to discharge, the percentage of women who breastfed was 88%, of whom 45% did exclusive breastfeeding. Only 35% of women who had a caesarean section adopted exclusive breastfeeding. In our experience, rooming-in was not associated with an increase in breastfeeding. We observed that both attendance to prenatal courses and the mother’s education level played a minor role in influencing the mother’s decision in breastfeeding A fairly high percentage of exclusive breastfeeding, 75%, was attained just in one hospital, where dedicated staff was deployed to encourage breastfeeding. The lowest percentage (12%) of exclusive breastfeeding was observed in a large private accredited health facility. Hospital presence of professionals trained in human lactation is a smart investment for society.

## 1. Introduction

Breast milk is a complete and balanced food for infants, with both short- and long-term positive effects on infant health. It has been shown that human milk, due to its bioactive composition, has microbiological, neuronal, endocrinological, and psychological benefits for the infant [1,2]. Furthermore, breastfeeding has demonstrated positive effects on child development [3].

In addition, breastfeeding has immediate and lasting benefits on maternal health, as demonstrated by several studies, which have shown a reduction in risk for some cancers, such as breast cancer, ovarian cancer, and endometrial cancer [4,5,6,7,8,9]. Benign breast disease does not usually interfere with a woman’s ability to breastfeed [10,11]. Similarly, polycystic ovary syndrome (PCOS) does not seem to be related to breastfeeding initiation and duration, while a high Body Mass Index (BMI) is negatively associated with breastfeeding [12,13,14,15]. It was also reported in the literature that breastfeeding lowers the risk of cardiovascular disease for both mother and child [16], as well as rheumatoid arthritis, and neurological diseases in the child [17,18,19].

The World Health Organization (WHO) and United Nations International Children’s Emergency Fund (UNICEF) recommend breastfeeding for the first six months of life and the complete weaning period, properly integrated with other foods, until two years old [20]. However, despite scientific evidence on its benefits, the adoption of exclusive breastfeeding in the European Region remains below the recommended level [21].

Several factors influence the success of breastfeeding, such as gestational age, parity, type of delivery, time from birth to first contact with the mother (for the newborn), participation in childbirth preparation courses, level of maternal education and socioeconomic status, employment, and quality of family relationships [22,23,24]. Several studies have also underlined the significant effects of psychological variables on breastfeeding exclusivity and duration [25]. More specifically, one of these variables is the presence of postpartum mood disorders [26,27].

Other factors that may influence the adoption of breastfeeding are related to the healthcare setting and to the social context. The type of hospital (private/public), their mission and size, or the number of deliveries and the prevalence of caesarean sections, could influence the choice of breastfeeding [28]. Social factors—including the prominence of extended family relations, acquaintances and professionals, media and websites as sources of advice concerning motherhood and breastfeeding—would also play a role.

In Italy, an Interdisciplinary National Technical Table for the promotion of breastfeeding was established in 2012. The main objective was to acquire information at a regional level that is useful for the planning of awareness programs and related strategies [29]. Among the main activities that this Technical Table recommends are included: the training of mothers, especially during preparation courses for childbirth and gynecological visits, professional updating of health care workers, as well as assistance to mothers, in order to start breastfeeding as soon as possible.

Our objective was to carry out an observation of the prevalence of breastfeeding in post-partum hospital stay, and the influence of factors that affect the success of breastfeeding and its modalities, in various types of hospital birth points. In Italy, a hospital birth point is a hospital ward, or unit, dedicated to the care of pregnancies and physiological infants, in which no less than 500 births occur per year, and that provides at least one delivery room and at least one adjoining operating room able for performing, at any time, a Caesarean section. A team with an obstetrician, an anesthesiologist, and a neonatologist is available 24 h a day.

Using a questionnaire administered to new mothers, we tried to characterize factors acting as barriers and facilitators in promoting breastfeeding in various types of hospital birth points in the province of Catania, in Sicily, Italy. The style of breastfeeding support adopted in the first days after birth is fundamental for the decision of women to continue, or not, with exclusive breastfeeding [30]. It is known that after discharge from the hospital the percentage of breastfeeding women decreases rapidly and does not increase in any case [31,32].

## 2. Materials and Methods

The study only involved birthing centers in hospitals. Delivery at home is not frequent in Italy; numbers are negligible (nor are available birthing places outside hospital). The survey was conducted in a variety of hospitals in the Province of Catania, Sicily, a region of Italy. The Health Education Unit in the local Provincial Health Authority (ASP), with the collaboration of the School of Hygiene and Preventive Medicine of the University of Catania, monitored the prevalence of breastfeeding at several birth points in Catania. The project was conducted during the years 2016–2018, at different times and in various hospitals. At the end of data collection, 3813 valid questionnaires were collected. 

The Province of Catania has public (National Health System—NHS) and private hospitals. The largest hospitals are located in the city of Catania. There are also a few community hospitals, serving smaller towns and communities in the province. The mothers were recruited in 7 out of 10 hospitals in this area, where 90% of the births of the province occur, according to data provided by the Health Department of the Sicilian Region for 2016–2018. 

For this study, participating hospitals were grouped according to their main mission, in terms of purpose or aim, and labeled as follows:

A—Community Hospitals: data were pooled from three small (less than 100 beds) community hospitals, each providing about 500 deliveries per year.

B—Public, Specialized Hospital: a large hospital, with a specialized Mother and Child Health Department, providing over 2000 deliveries per year.

C—Public, Emergency Hospital: a large hospital, well known for emergency services, including public helicopter rescue service and an operating base for public ambulances, with over 1000 deliveries per year.

D—Public, Teaching Hospital: a teaching and research academic hospital with two large hospital centers, each providing childbirth services, with over 3500 deliveries per year.

E—Private Hospital: the largest of a few private health facilities in the area, accredited with the Regional Health Department, authorized for childbirths, with over 800 deliveries per year.

Hospitals B, C, and D are accredited according to criteria of availability of high technology, high organizational specialty, and professional medical nursing, with gynecological and neonatal intensive care units.

### 2.1. Questionnaire

An online questionnaire, created ad hoc (Google Forms, Google LLC, Mountain View, CA, USA), was used and administered using a tablet, by the midwife to the mother, after discharge (Computer-Aided Personal Interview). The purpose and modality of the study was explained in advance to the mother, before enrollment. Participation in the study was voluntary and processing of mother and baby’s data was in accordance with the Privacy Acts (General Data Protection Regulation—GDPR, EU Regulation 2016/679). 

The questionnaire presented 16 questions in 2 groups: general questions concerning the mother and the delivery; questions concerning feeding of newborn, education, information provided to the mother before delivery, and rooming-in of the newborn.

Education level was classified as: primary = elementary and middle schools; secondary = high school; tertiary = university bachelor’s/master’s/doctoral degree. 

Feeding was classified as exclusive breastfeeding, if only breast milk was used and no other nutrients were provided; artificial, if the newborn had not been attached to breast, and only baby formula or glucose gel had been provided, and mixed if both breastfeeding and artificial feeding had occurred.

Data were gathered in a Google Sheet document and transferred to a Microsoft Excel worksheet (Microsoft, Redmond, WA, USA) for subsequent analysis. The percent coverage of this survey was evaluated dividing the number of women enrolled during the enrolment time interval in each hospital, by an estimate of the deliveries occurring in the same interval based on yearly deliveries, obtained from the Department of Epidemiology of the Regional Government.

### 2.2. Statistical Analyses

Data were analyzed using Analyse-it for MS Excel 5.40 (Analyse-it Software Ltd., Leeds, UK). Results were reported as mean, standard deviation, and min–max range for numerical variables, and as percentages for categorical variables. Confidence Intervals at the 95% level (95% CI) were also computed for selected results. Bivariate and multivariate tables were analyzed with the chi-square test for frequencies, and the resulting probability level was reported whenever smaller than the set level *p* = 0.05. Analysis of Variance (ANOVA) was also employed at same significance level.

## 3. Results

Table 1 details distinctive features obtained from the reported number of valid questionnaires, in total and by type of hospital.

The average maternal age was 31.3 ± 5.8 years (range 15–58). The mean age was lower in type a hospitals, located outside the large city, compared to city hospitals. The observed incidence of caesarean deliveries, which was overall similar to what was observed in this region, was higher in hospitals C and E.

About half of the enrolled women were at their first delivery; rooming-in of the newborn in the same room with the mother was available in all facilities and adopted in most cases (between 66% and 100%). 

Table 1 also reports that only 31% of women had attended a prenatal course for birth preparation (14%–38% in various types of hospitals); therefore, 69% of women did not attend a prenatal course.

**Figure 1:**
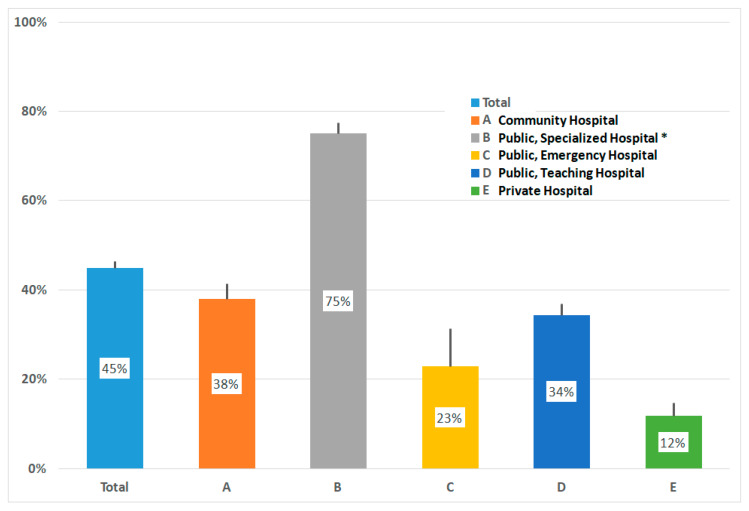
Percentage of exclusive breastfeeding; vertical lines indicate 95% CI. * Significantly different from A, C, D, E, *p* < 0.0001 chi-square test.

From birth to discharge, the percentage of women who breastfed was 88% (3464/3813); of these, 45% (1713) provided exclusive breastfeeding, whereas 46% (1751) did mixed breastfeeding, where in most occurrences (1481) baby formula was fed too; while in 240, glucose gel (200 mg/Kg) was used, and 30 were unspecified. In the remaining 349 babies, baby formula only was used, except for three cases, where glucose gel was given. Reasons reported for inability to breastfeed were severe malformations, hospitalization in Neonatal Intensive Care Unit (NICU), and maternal illness. Considering that mixed feeding is considered suboptimal, just the percentage of exclusive breastfeeding carried out in the different hospitals up to discharge is reported in Table 1 and Figure 1. Exclusive breastfeeding was observed in 45% of all women, and variations between 12% and 75% were observed (95% Confidence Limits are marked in figures). It was just in Hospital B, where a specialized Mother and Child Department operates, that exclusive exceeded mixed breastfeeding (75% vs. 20% respectively). 

**Figure 2:**
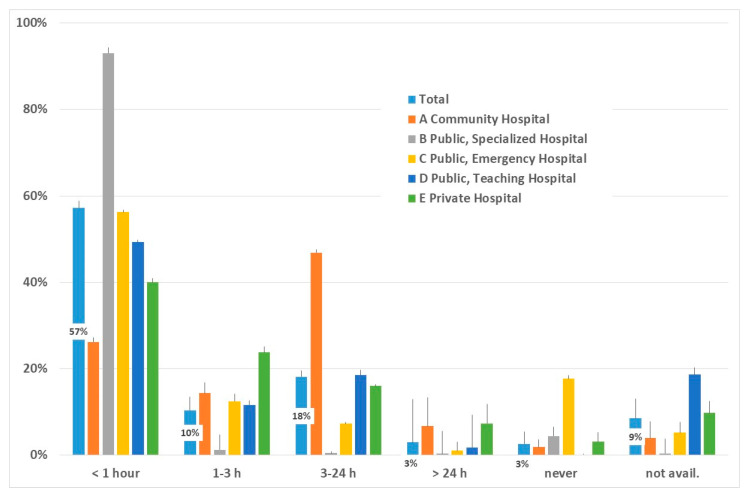
Time of start of breastfeeding after delivery; percentages are indicated for columns representing total results. Vertical lines indicate 95% CI.

Figure 2 shows the percentage of infants attached to the breast within the first hour after delivery and in subsequent periods, overall and in different types of hospitals. The start of breastfeeding occurred, in most hospitals, within the first hour (57%), except for community hospitals, where breastfeeding was delayed. Further in time, breastfeeding started in 10% of cases within the first 3 h, in 18% within 24 h, in 3% after the first 24 h, and 3% were never attached to the breast; information was not available in 9%.

**Figure 3:**
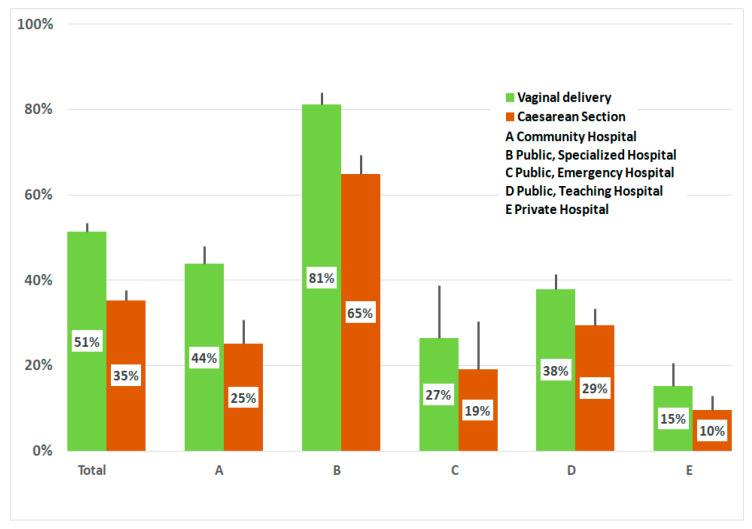
Percentages of exclusive breastfeeding in vaginal and caesarean delivery in various types of hospitals. Vertical lines indicate 95% CI.

The decision to breastfeed, from birth to discharge, appears to be affected by the type of childbirth (Figure 3). Just 35% of women who had caesarean sections adopted exclusive breastfeeding, compared with 51% of those who had vaginal delivery (*p* < 0.001, chi-square test). It was only in Hospital B where we found a high percentage of exclusive breastfeeding achieved, both in women who gave birth vaginally (81%) and in women who had a caesarean section (65%).

**Figure 4:**
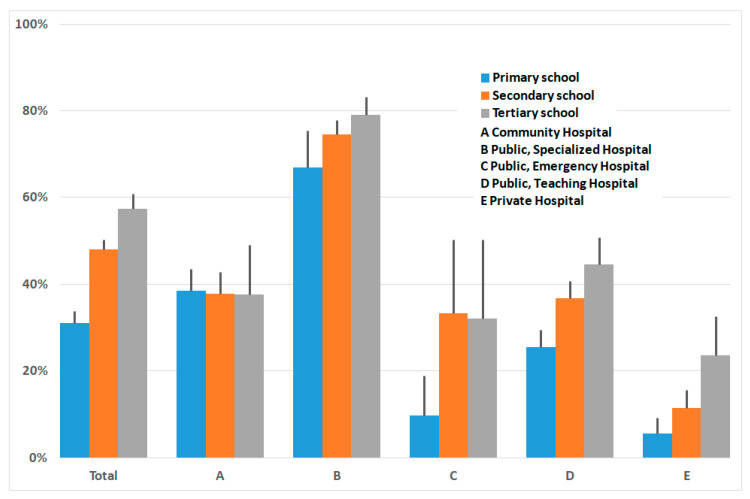
Percentage of exclusive breastfeeding by education level attained by the mother, overall and in various hospital settings. Vertical lines indicate 95% CI.

Looking for a relationship between breastfeeding and the mother’s education levels (Figure 4), we observed that the percentage of women who breastfed before discharge increased, overall, with education level, from 31% in primary to 48% in secondary and 57% at the tertiary level. In detail, it appeared evenly distributed in type A and atypical in Hospital C, where a large uncertainty was noticeable due to a relatively smaller number of questionnaires.

**Figure 5:**
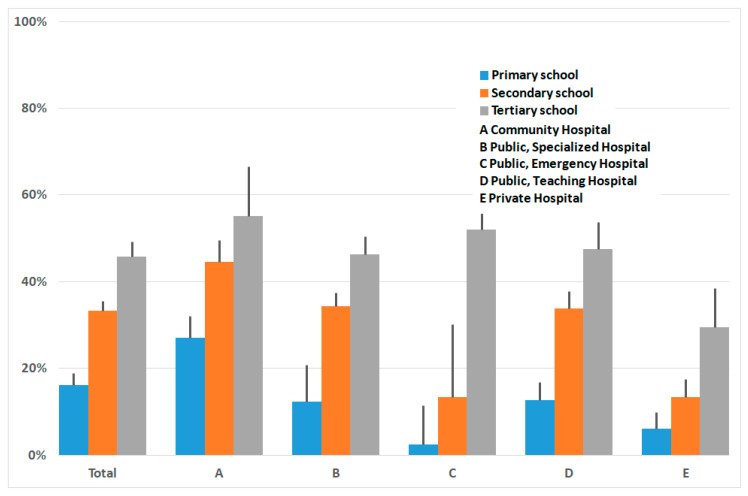
Percentage of women who had attended birth preparation courses, by education level, overall and in various hospital settings.

The percentage of women who were exclusively breastfeeding was 53% among women who had attended prenatal courses (1170), and 42% among women (2610) who had not attended the courses (*p* ≤ 0.0001). Attendance to birth preparation courses was related to schooling levels (Figure 5).

The percentage of mothers who asserted that they had received information on the importance of breastfeeding was high (89%), with limited variation (79%–93%) across hospitals (Table 1). 

**Figure 6:**
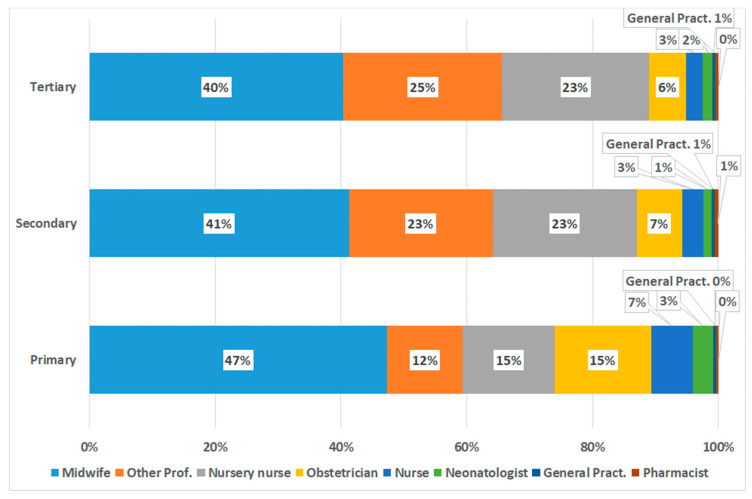
Leverage of various professionals in providing information to mothers, according to women’s levels of education. Other Prof. = other professionals encountered. General Pract = General Practitioner (NHS Primary Care Physician).

**Figure 7:**
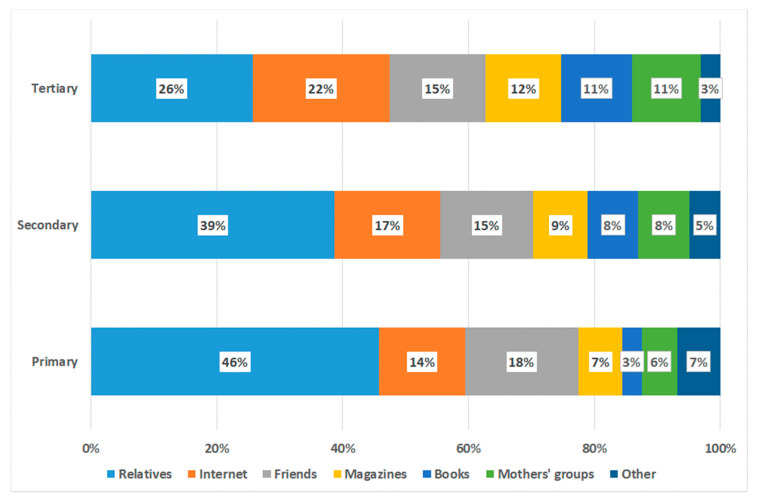
Other sources of information for mothers, according to women’s levels of education.

Figure 6 and Figure 7 report the source of information about breastfeeding, from professionals and from other sources of information, by education level. Among professionals, midwives and other professionals exerted most influence. Among other sources, relatives prevailed, although with a relevance declining with increasing education. Besides relatives, the internet and friends contributed the most, about evenly by education level.

## 4. Discussion

The decision to breastfeed was influenced by a convergence of factors. No one had previously compared the different health facilities to evaluate if the different missions of these facilities, and the related hospital organizations, could influence the decisions of women who had given birth to start breastfeeding, in the period between birth and discharge. 

The average hospital stay after childbirth is 3–4 days (considering both the vaginal delivery and caesarean sections); therefore, it is important to start breastfeeding from the very beginning.

The wide variation observed between hospitals, in the percentage of women who chose exclusive versus mixed breastfeeding, or no breastfeeding at all, is something to consider. The minimum level is observed in a large private accredited health facility, where mixed or artificial breastfeeding might be actively proposed. Therefore, despite the greater comfort and human touch available in private hospitals, this aspect is not privileged, even with planned delivery and the convenient environment of single rooms.

The hospital dedicated to the emergency provided a very low rate of response to the questionnaires, even though the number of deliveries was considerable. This might indicate a lack of interest in the subject. It would seem that this hospital did not invest in communicating with mothers. On the other hand, during the observation period, in another hospital, a research project allowed the hiring of a professional dedicated to recording the timing of infant attachment to the breast. This intervention brought remarkable results in the percentage of adhesion to exclusive breastfeeding.

It is important to offer to all women the opportunity to try breastfeeding immediately after childbirth; it is well reported that if breastfeeding does not start within a few hours after childbirth, the mother will never breastfeed or will stop early. It is very important that breastfeeding start within 24 h of delivery. We observed that 85% of children were attached to their mothers’ breasts within the first 24 h. Those who do not take the breast within the first day of life will very rarely do so in the future, and the first three hours are essential to ensure the continuation of exclusive breastfeeding [22,30,33,34].

Vaginal delivery stimulates breast milk secretion and is accompanied by less painful breastfeeding. These factors could explain the results related to higher, exclusive breastfeeding after vaginal delivery compared to caesarean section. Comparing breastfeeding rates after vaginal deliveries or caesarean sections, the only hospital where the percentage is reversed, in favor of the caesarean section, is a private hospital, where most deliveries are programmed in advance. It is regrettable to point out that, despite the regional policy equating the reimbursement of vaginal childbirth to that for the caesarean section, hoping to obtain a boost for an increase in vaginal births, this has not yet proved to be within the limits expected for the appropriateness of care. It is considered that a 20% percentage for caesarean sections can sufficiently account for cases where the caesarean section is the correct indication. An abnormally high percentage of caesarean sections occurred in the emergency hospital, where the two modalities of delivery attain equal percentages.

It is reported that rooming-in increases breastfeeding initiation [35,36,37], but the effects of rooming-in on breastfeeding remain uncertain [38,39]. In our experience, rooming-in is not capable of causing an increase in breastfeeding by itself. The rate of exclusive breastfeeding is low in a private facility with 100% rooming-in, private rooms, free access for family and visitors, hotel level comforts (E, 12%); it is quite different in two facilities with similar use of rooming-in (B, 75% breastfeeding vs. 96% rooming-in; D, 34% breastfeeding vs. 96% rooming-in). There is no correlation between rooming-in and the percentage of breastfeeding women.

Attendance at prenatal courses is claimed to encourage breastfeeding [40]; we observed that it plays a limited role in influencing the mother’s decision on breastfeeding with a difference of just 11%. It is possible to identify, in the education level, a factor that influences the type of choice made by the mother. Education levels influence the participation of women in prenatal courses, the type of information source, and the percentage of exclusive breastfeeding. We found that a higher level of education of the mother corresponds to a higher percentage of exclusive breastfeeding and, therefore, should guarantee better health for the child. 

We also observed that in almost all different types of hospitals, lower education levels are accompanied by lower rates of breastfeeding, although this difference did not occur in community hospitals. Since the educational level is related to breastfeeding behavior, if we want to have an effect on neonatal health, we must intervene in the mother’s education, as a public health problem, and targeted action should be proposed.

Although information about breastfeeding was provided in all hospitals to the majority of women, this was ineffective to sustain breastfeeding. The adoption of exclusive breastfeeding in Hospital B could be explained by the presence of a dedicated person providing the information.

Analyzing the possible sources of information, we observed that midwives have a prevailing role, regardless of the education level of the mother, among the health professionals. The weight of information given by medical doctors is inversely proportional to the level of education. In any case, the doctor’s influence (obstetrician, pediatrician, or family primary physician) has a marginal role or none. Who exactly the “other professionals” intervening might be is not detailed from the data. However, considering that their influence increased with education level, we might guess that it was friends; thereby, explaining an influence overtaking that of nursery nurses, gynecologists, or pediatricians. 

Looking at any source of information—other than healthcare professionals—it can be seen that, according to the answers given, family plays the predominant role, although with relevance declining with increasing education. The internet supplanted TV, newspapers, magazines, and books, which remain a source of information only for the highest education levels, where books are surpassed by mommy groups. 

Neither family members nor the partners proved to be supportive, probably due to the local habit of excluding them from all training programs. It is described in the literature that the involvement of fathers has great effectiveness in material and psychological support to ensure exclusive prolonged breastfeeding [41]. This never happened in the hospitals surveyed. 

These results allow suggesting programs of effective intervention to promote breastfeeding immediately after childbirth. The goal of hospitals should be to increase the percentage of infants who start life being breastfed. It is reported that the duration of breastfeeding is determined by the initiation of breastfeeding in the first hours after delivery.

The only variable that has led to a substantial change in the proportion of women who breastfed exclusively between the various hospitals was the presence, during postpartum, of a nursery nurse specialized in infant care and maternal pedagogy. A lactation consultant after birth seems to be more helpful than prenatal course.

To achieve a higher overall percentage of breastfeeding women, due to a lack of sufficient funds, training should focus on women with lower levels of education. This subgroup, which is numerically important in all hospitals, is the one that could benefit the most from information from the staff in the hospital, taking into account the previous misinformation on the subject. Therefore, selected training for these women might turn out more useful to reduce disadvantage coming from low education levels and, consequently, minor autonomy of choices.

The model of one-to-one consultation during postnatal hospitalization seems to be the most effective, as previously reported in a pilot study of a breastfeeding-support intervention [42]. A nurse dedicated to postpartum care has to teach the correct techniques and manoeuvers that can promote breastfeeding, but should also provide the new mother with all of the information regarding the benefits for the child, and the short- and long-term benefits for the mother. She will also be able to provide information on three areas in which breastfeeding can reduce morbidity: risk of infection, cancer, and obesity. Breastfeeding promotes the development of indigenous flora; thus, protecting infants from infection [43,44,45]. The risk of opportunistic infections contracted in birth points is greater in infants fed on formula than in breastfed infants. Despite good compliance with hand hygiene procedures and delivering care routines, artificial breastfeeding represents an increased risk to the exposure of contaminated objects from the hands of carers, as well as direct contact with the hands, which are the most important vehicles of microorganisms in hospitalized patients. 

Moms must also be informed that it has been proven that breastfeeding reduces the risk of cancer, such as breast, ovarian, and endometrial cancer. Moreover, breastfeeding duration has a stronger protective effect. The presence of benign breast cancer does not usually interfere with the woman’s ability to breastfeed [10]. Breastfeeding is not a risk factor for a hepatitis C virus mother- to-child transmission [46]. It is suggested to refrain from breastfeeding only if the nipples are cracked or bleeding. 

The healthcare worker dedicated to supporting the new mother can also give information on proper diet and hygiene through the transmission of healthy eating habits, which can promote lactation. Pregnant women should increase the nutritional quality of breakfast, reducing the consumption of snacks and increasing the consumption of fresh fruit. The adoption of regular meals and an increase in the quality of diet, as well as promoting the growth and physical development of the newborn, reduce the prevalence of obesity and glycemic problems often related to pregnancy [47]. Breastfeeding reduces obesity in mother and child [48].

Finally, this professional, giving complete and accurate information, contributes toward changing breastfeeding practices and teaching mothers the principles of childcare. Furthermore, it is also important to provide positive psychological support to mothers who have perinatal depression and who intend to breastfeed, by also involving a psychologist who specializes in postpartum interventions, and who can work together with the nurse [25,26,27].

Since increasing the percentage of breastfeeding women is a success for the whole society, the National Health System must try to identify and implement all of the interventions that can support breastfeeding [49,50]. The use of the internet for the spread of good practices is important because it reaches mothers regardless of the level of education. The presence in the hospital of a professional trained in human lactation is a smart investment in providing benefits for society [51].

Considering the data presented, it is clear that each kind of hospital can contribute to the increase in exclusive breastfeeding only if they manage to have staff dedicated to supporting breastfeeding in the early stages after birth. The intervention will be even more effective if it is diversified, according to the characteristics of the women who have recently given birth.

## 5. Conclusions

For breastfeeding, the hospital’s vocation is decisive, and children born in a baby-friendly hospital are more likely to be breastfed for a longer time. The emergency hospital did not pay particular attention to this aspect. The private facility, although equipped with hotel comforts and access facilities for maternal support, did not come out with high percentages of breastfeeding. Rooming-in was useful, but not enough to increase the percentage of breastfeeding. The type of delivery determined a difference in only 15% of cases of exclusive breastfeeding. The main factor associated with the continuation of exclusive breastfeeding is the first early feeding, which stimulates physiological, affective, and psychological factors in the mother.

There were no differences in the sources of information between the different hospitals. Differences were observed between different levels of education. In most mothers, of all hospitals, midwives and nurses, between healthcare workers, provided knowledge of the benefits of breastfeeding. Among non-health sources, besides relatives, internet and mommy groups won out versus books, television, or newspapers.

The highest result in breastfeeding percentage was observed in the hospital that invested in healthcare personnel dedicated to recording cases of newborns, their frequency of attachment to the breast, and providing help and information on breastfeeding.

The cultural differences, already described, allow identifying a sub-group in which greater benefits can be obtained: prenatal courses or nurse information, focused at women with primary levels of education, can improve the percentage of breastfeeding.

Finally, it is considered useful to suggest the use of the internet for the spread of good practices, since it appeared to reach mothers, regardless of education level.

Further studies should specifically address the influence of organizational factors in the uptake of exclusive breastfeeding in hospital birth points in Italy, especially considering social differences in society.

## Figures and Tables

**Table 1:** Characteristics of Women from Questionnaires.

Data	Total	Community Hospital (A)	Public, Specialized Hospital (B)	Public, Emergency Hospital (C)	Public, Teaching Hospital (D)	Private Hospital (E)
No. of questionnaires	3813	789	1193	96	1285	450
% coverage *	38	70	32	32	23–36 **	28
Age (yrs., mean ± std. dev.)	31.3 ± 5.8	29.7 ± 6.0 *§*	31.9 ± 5.6	31.4 ± 6.1	32.1 ± 5.5	31.3 ± 5.7
Age range (years, min–max)	15–58	16–52	15–50	21–58	17–48	15–44
Education level						
primary %	15	34	4	6	15	14
secondary %	53	53	56	22	53	46
tertiary %	31	13	40	72	32	40
Caesarean Section %	39	29	37	49	39	61
Primiparae %	48	43	52	57	48	41
Rooming-in %	91	66	96	81	99	100
Birth preparation courses %	31	38	36	19	29	14
Received information on breastfeeding in hospital %	89	93	99	90	79	86
Exclusive Breastfeeding %	45	38	75 ^§§^	23	34	12

* Coverage estimated from (number (no). of questionnaires)/(approximate no. of deliveries in the time interval of administration of questionnaires). ** In two different locations. *§* significantly lower than in type B, D, E of the hospital (*p* < 0.0001, ANOVA). ^§§^ significantly higher than in type A, C, D, E hospital (*p* < 0.0001, chi-square test).
